# Reducing contrast agent residuals in hospital wastewater: the GREENWATER study protocol

**DOI:** 10.1186/s41747-023-00337-w

**Published:** 2023-05-04

**Authors:** Moreno Zanardo, Andrea Cozzi, Rosanna Cardani, Laura Valentina Renna, Francesco Pomati, Luigi Asmundo, Giovanni Di Leo, Francesco Sardanelli

**Affiliations:** 1 Department of Biomedical Sciences for Health, Università degli Studi di Milano, Milan, Italy; 2 Unit of Radiology, IRCCS Policlinico San Donato, San Donato Milanese, Italy; 3 Biobank BioCor, IRCCS Policlinico San Donato, San Donato Milanese, Italy; 4 ArsChemica S.R.L., Caselle Landi, Italy; 5 Postgraduation School in Radiodiagnostics, Università degli Studi di Milano, Milan, Italy

**Keywords:** Contrast media, Gadolinium, Iodine, Outpatients, Urinalysis

## Abstract

The potential enviromental impact of iodinated (ICAs) and gadolinium-based contrast agents (GBCAs) have recently come under scrutiny, considering the current nonselective wastewater treatment. However, their rapid excretion after intravenous administration could allow their potential recovery by targeting hospital sewage. The GREENWATER study aims to appraise the effective quantities of ICAs and GBCAs retrievable from patients’ urine collected after computed tomography (CT) and magnetic resonance imaging (MRI) exams, selecting ICA/GBCA per-patient urinary excretion and patients’ acceptance rate as study endpoints. Within a prospective, observational, single-centre, 1-year framework, we will enrol outpatients aged ≥ 18 years, scheduled to perform contrast-enhanced CT or MRI, willing to collect post-examination urine in dedicated canisters by prolonging their hospital stay to 1 h after injection. Collected urine will be processed and partially stored in the institutional biobank. Patient-based analysis will be performed for the first 100 CT and 100 MRI patients, and then, all analyses will be conducted on the pooled urinary sample. Quantification of urinary iodine and gadolinium will be performed with spectroscopy after oxidative digestion. The evaluation of the acceptance rate will assess the “environmental awareness” of patients and will aid to model how procedures to reduce ICA/GBCA enviromental impact could be adapted in different settings.

**Key points**

• Enviromental impact of iodinated and gadolinium-based contrast agents represents a growing point of attention.

• Current wastewater treatment is unable to retrieve and recycle contrast agents.

• Prolonging hospital stay may allow contrast agents retrieval from patients’ urine.

• The GREENWATER study will assess the effectively retrievable contrast agents’ quantities.

• The enrolment acceptance rate will allow to evaluate patients’ “green sensitivity”.

## Background

Imaging techniques, such as contrast-enhanced computed tomography (CT) and contrast-enhanced magnetic resonance imaging (MRI), represent fundamental tools in almost every clinical setting [1, 2]. Contrast agents (CA) for CT are iodinated compounds (ICAs), the most widely used pharmaceuticals for intravascular administration [3, 4]. Most ICAs are derivatives of the triiodobenzoic acid, largely eliminated via urinary excretion without being metabolised, generally within 24 h after administration [4–7]. Conversely, contrast-enhanced MRI uses gadolinium-based CAs (GBCAs), chelates of the trivalent gadolinium ion with polyaminocarboxylic acids. Chelation ensures that the toxic effects of the free gadolinium are avoided and allows for the excretion of GBCAs without metabolisation [2, 8–10]. ICAs and GBCAs are dosed according to the patient’s body weight or with fixed doses [11], as attempts to further optimise the volume of injected CA [11–14] have still to gain widespread adoption.

Adverse effects of ICAs are rare and mostly emerge as short- and medium-term consequences, with hypersensitivity reactions and contrast-induced nephropathy [15–17]. Conversely, adverse effects of GBCAs may present as medium- and long-term consequences, specifically nephrogenic systemic fibrosis [18]. Gadolinium retention in the brain and other organs has been reported as well [18, 19].

Overall, ICAs and GBCAs have a generally high safety profile, with a positive tradeoff between clinical benefits and adverse effects. However, there is growing attention about potential effects of CAs that enter aquatic environments from hospital sewage [4, 9, 20–23], as also highlighted by European initiatives focused on the reduction of pharmaceutical contamination of ecosystems.

The chemical stability of ICAs and GBCAs explains the abundant literature that documents their presence in surface water, groundwater, and even in drinking water [3, 4, 8–10, 21–25], especially near hospitals and wherever drinking water is produced from raw water resources. Kormos et al. [3] reported that as much as 46 different transformation products of ICAs are detectable in aquatic environments before wastewater treatment, with at least 26 of them persisting in effluents from treatment facilities. As the environmental half-life of GBCAs can reach 130 days [10], anthropogenic gadolinium has been detected even in sewage treatment plants of rural areas [26] and attributed to patients who return home after having undergone MRI as outpatients. Several aspects of this scenario remain to be investigated, including the environmental fate of GBCAs, the mechanisms of their bioaccumulation, and their biochemical effects [23].

Indeed, a clear appraisal of the long-term enviromental impact of ICAs and GBCAs is still lacking [4, 9, 21]. ICAs presence in environmental waters favours their reaction with other organic and inorganic compounds, leading, under laboratory stressed condition, to the formation of known iodinated degradation by-products. In real life conditions, the amount of such degration by-products, if any, is aspected to be at trace levels. Overall, the magnitude of this problem is still to be ascertained [4, 21]. Potentially, the effects of water microcontamination by GBCAs also relate to the formation of less stable gadolinium chelates and to gadolinium bioaccumulation [9], but the even smaller scale of this phenomenon implies that it is equally harder to precisely model its relevance for human health. In any case, even considering these knowledge gaps, the poor retrievability of ICAs and GBCAs from conventional wastewater treatment plants underlines the need to define and implement strategies to minimise the release of ICAs and GBCAs in the acquatic enviromentt [4, 10, 23–27]. The ICAs and GBCAs rapid excretion profile could allow for a considerable recovery by specific targeting of hospital sewage, for example if outpatients being administered CAs could be kept in the facility long enough to allow for the urinary excretion of a sizable quantity of these CAs.

To provide preliminary data about the potential reduction of CAs residuals in wastewater and to estimate CA excretion in the first hour after administration, the GREENWATER (reducinG contRast agEnts’ rEsiduals iN hospital WAstewaTER) study aims to prospectively monitor the quantity of retrievable ICAs and GBCAs from urine collected from outpatients within an hour from administration.

## Methods

### Study design and endpoints

This prospective monocentric observational study has been registered on Zenodo (https://zenodo.org/record/7800690) and has been approved by the local ethics committee (IRCCS Ospedale San Raffaele, Milan, Italy) on May 11, 2022, with control number 53/INT/2022. Enrolment is ongoing at the Radiology Unit of IRCCS Policlinico San Donato (San Donato Milanese, Italy). Urinary excretion of CAs per patient within 1 h—in relation to patients’ age and sex, season, and other covariates—will be the primary study endpoint, while study acceptance by the patients, measured as a rate on the total, will be the secondary endpoint.

Considering the high variability of inpatient conditions as well as regulatory issues that hinder straightforward collection, storage, and analysis of biological samples from patients with potential infectious diseases, this preliminary study will enrol only outpatients. Inclusion criteria will be as follows: outpatients (males or females) scheduled to perform a contrast-enhanced MRI or a contrast-enhanced CT for any reason and age ≥ 18 years. Exclusion criteria will be as follows: inability to provide specific informed consent, unwillingness to extend the observation time after examination to 60 min after administration, unwillingness to provide the requested urine sample, the presence of known infectious disease in the last 2 months, and the presence of signs and symptoms of ongoing infectious diseases.

### Study workflow

**Fig. 1:**
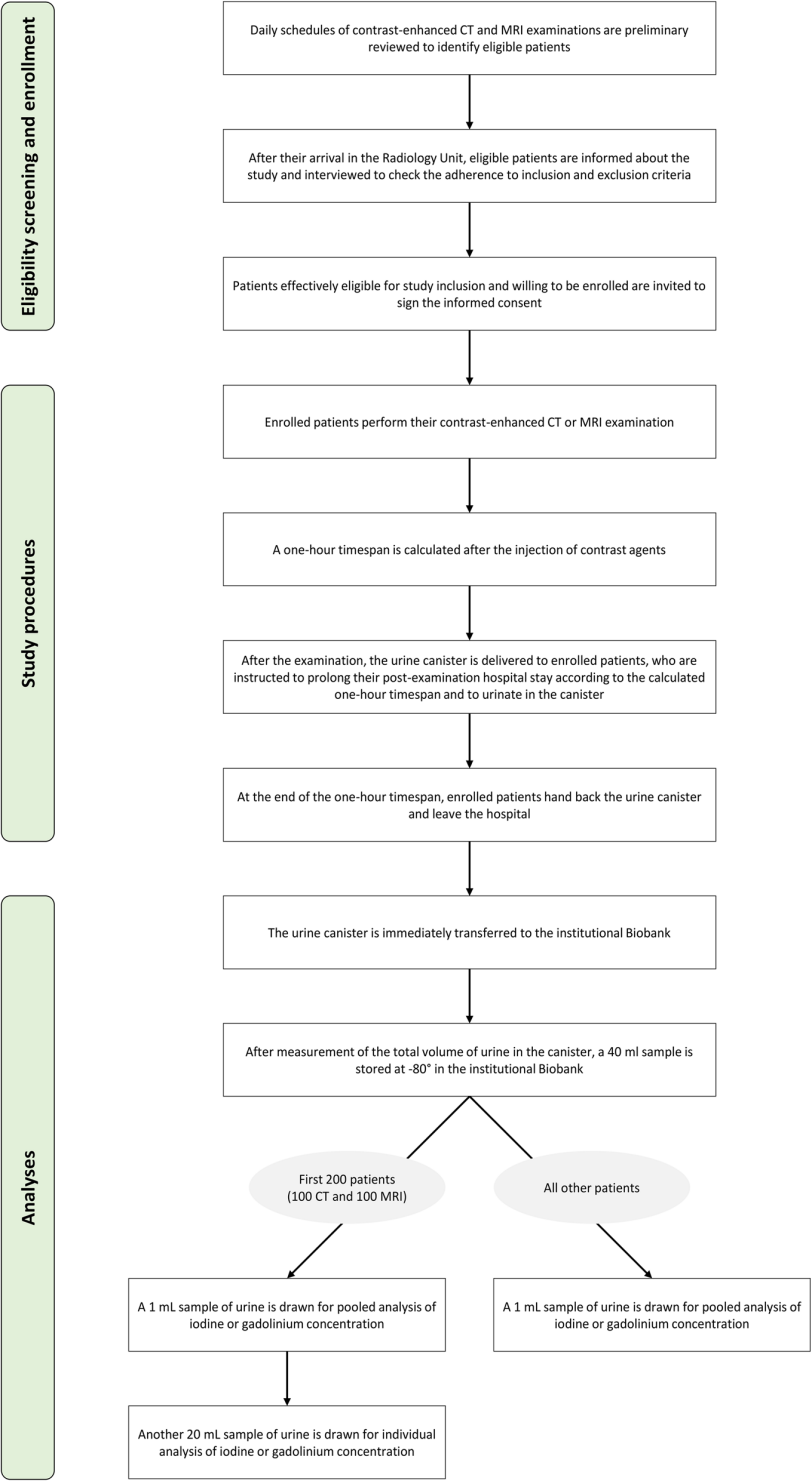
Study workflow

As detailed in Fig. 1, patients eligible for enrolment will be informed about the study aims and design. For each eligible patient, data on age, sex, and type and time of scheduled examination will be recorded to estimate the possible impact on the two endpoints by these variables. Of note, eligible patients will be considered according to two age groups (18–65 and > 65 years). Enrolment will follow a nearly 1:1 ratio between these two age categories.

Eligible patients willing to participate in the study will sign the specific informed consent of the study and that of the institutional biobank. After standard anamnesis, all enrolled patients will undergo their scheduled contrast-enhanced examination (CT or MRI) without any modifications to the current clinical protocols. For each patient, serum creatinine levels, estimated glomerular filtration rate, contrast agent molecule, concentration, dose, and injection rate will be recorded alongside examination protocol, type, and diagnostic purpose. Considering the results of previous studies and environmental projects which investigated the compliance of patients to different urine collection methods [22] and the aforementioned rapid excretion profile of ICAs and GBCAs, we opted to target the first urinary excretion after contrast-enhanced CT and MRI. This arrangement represents a compromise between the amount of collaboration required from the patients and the ability to collect urine samples during the CA excretory phase peak. Thus, after the examination, the usual observation time of approximately 30 min will be extended to 45–50 min, with a total timespan after CA administration of 60 min. During this time interval and specifically before leaving the hospital, enrolled patients will be required to urinate in a urine canister.

**Fig. 2:**
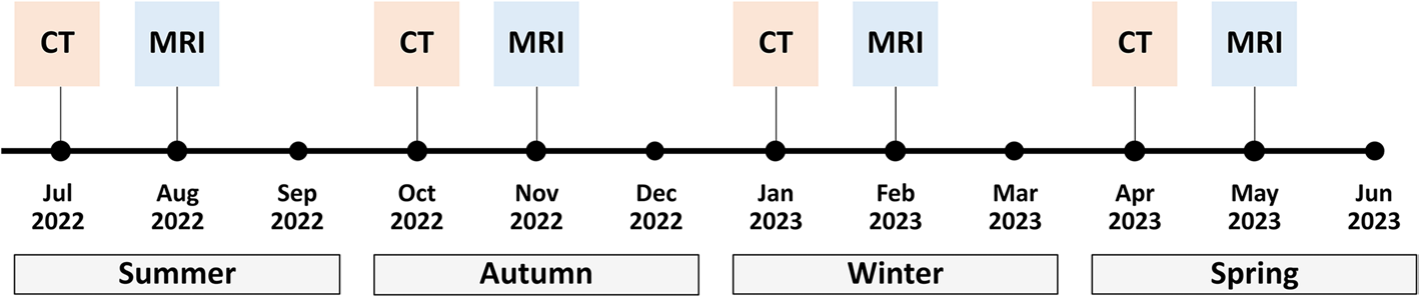
Timeline of patient enrolment according to seasons

In order to account for seasonal variability of scheduling and climate, the enrolment will be season synchronised and will span over 12 months from July 2022 onwards, as detailed in Fig. 2. In each season, the first month (July 2022, October 2022, January 2023, April 2023) will be reserved for the enrolment of patient scheduled to undergo contrast-enhanced CT, while the second month (August 2022, November 2022, February 2023, May 2023) will be reserved for the enrolment of patients scheduled to undergo contrast-enhanced MRI. If the number of planned enrolments per month will be reached, no enrolments will be done in the third month of each season. Conversely, supplementary enrolments will be planned in the third month of each season.

### Sample size

The sample size was obtained considering an average number of 16 outpatients per day scheduled at our institution, an average of 20 working days each month, and an expected acceptance rate of 30%. Thus, 100 patients per month are expected to be enrolled, for a total of 800 patients over 12 months, *i.e.,* 200 patients per season. Patients will belong to the CT group (400 patients) or to the MRI group (400 patients), and each main group will be divided into 16 subgroups of 25 patients according to sex, age, and season, for a total of 32 subgroups, as follows:

1. males aged 18–65 (one group for each season for each CA);
2. females aged 18–65 (as above);
3. males aged > 65 (as above);
4. females aged > 65 (as above).

Although the sample size was not based on formal calculations, 25 patients per each subgroup are about two times the recommended number per subgroup for multivariable regression analysis, which will be used, as explained below, to model the relationship between several covariates and CA concentration in the urinary samples. Indeed, the Widrow-Hoff learning rule suggests the presence of ten data (patients) for every subgroup [28].

### Urine samples treatment and analysis

Collected urine will be immediately transferred to the BioCor institutional biobank, where samples will be processed according to the standard operating procedures which regulate the biobank activities. After measuring the total volume of urine collected from each patient, 40 mL of urine will be centrifuged at 2,500 × *g* for 5 min at room temperature and then aliquoted and stored at -80 °C. To ensure appropriate pseudonymisation, all aliquots of the same patient will be associated with the patient’s unique alphanumeric identification code, and the traceability of the aliquots will be managed through a dedicated software using the barcode scanning technology.

For the overall analysis related to the primary endpoint, we will obtain for each of the 32 subgroups of patients a pooled sample of urine by pooling 1 mL of urine from each patient of the subgroup. These 32 pooled samples will be packaged and transported in dry ice to ArsChemica S.R.L. (Caselle Landi, Italy) according to the International Air Transport Association regulations. A data logger device will be used to monitor the shipping temperature. Moreover, for the first 200 enrolled patients only (100 CTs and 100 MRIs), a patient-based analysis of urinary CA concentration will be carried out on another 20-mL sample that will be packaged and shipped for analysis as described before.

### Chemical analysis

All standard chemical analyses for the measurement of iodine or gadolinium concentration will be performed at ArsChemica S.R.L.

Quantification of urinary gadolinium and iodine will be performed using a two-step procedure. The first step will be a microwave digestion in very hard oxidative conditions; the second one will be the analysis by inductively coupled plasma optical emission spectroscopy. The oxidation will be carried out in a closed vessel microwave digester, dissolving 200 µL of urine in 10 mL of aqua regia. The same mineralisation pathway will be used regardless of the type of CA, but the sample will be further diluted according to its initial concentration to fall within calibration range. The quantitation range spans linearly in the 0.25–10 mg/L concentration range, and the calibration plot has been calculated through a least squares model with five concentration levels each one three times replicated. The preferred emission lines of the atomic elements are as follows: 335.047 nm and 310.050 nm for gadolinium and 178.276 nm and 183.038 nm for iodine. The matrix effect will be studied with spiked urine sample at 0.5–1 mg/L, and for both elements, spectral interferences will be always under control. The iodine carry-over effect will be reduced almost to zero by adding to the working standards a washing solution of ammonium hydroxide and 0.5% of 2-butanol; in these conditions, recovery is always between 80 and 120% of the added theoretical amount. The method is devised to be repeatable within 5%.

### Statistical analysis

For the overall analysis related to the primary endpoint, mean CA content will be measured for each of the 32 subgroups (2 CA types × 4 seasons × 2 genders × 2 age groups) on the pooled urinary sample and reported as mean and standard deviation or median and interquartile range according to data distribution. For the analysis related to the secondary endpoint, the acceptance rate will be calculated over the whole sample and in each of the 32 subgroups, investigating potential differences in age, timing of the examination, and other retrieved patient characteristics with nonparametric tests, as appropriate according to variable distributions. For the first 200 enrolled patients, the influence of patient-specific and examination-specific covariates on the urinary excretion of CAs within 1 h will be investigated with multivariable regression analysis, independently for the ICA group and the GBCA group.

## Expected results and future impact

The results of this study will help to assess how much iodine and gadolinium could be prevented to enter unmonitored sewers by implementing a small extension (up to 1 h) of the observation period after CA injection for contrast-enhanced CT and MRI examinations of outpatients. Collected data, in particular the amount of retrieved iodine and gadolinium in relation to the amount of iodine and gadolinium administered (also using an intention-to-treat approach), will inform the design of future studies.

This hospital-centred study differs from other studies that involved the use of urine bags up to several days after examination, as conducted in the Netherlands (the “Brede Proef Plaszakken” project, https://van-waarde.com/portfolio/brede-proef-plaszakken/) and Germany (the “MERK’MAL” project, https://merkmal-ruhr.de). As this study collects each patient’s urine during 1 h after CA injection, it does not require any further collaboration from the patient outside the prolonged in-hospital time.

We also expect that results of this study will help to start a comprehensive evaluation of the role of onsite wastewater treatment solutions managed by hospitals. While the feasibility of such solutions has been proved [27], benefits and costs of their routine implementation remain to be ascertained. Early treatment of hospital wastewater could extend potential benefits in overall sustainability of CAs, also improving economic efficiency and global resource management through recycling iodine and gadolinium, especially in light of the scarcity of gadolinium reserves and the ecological impact of GBCAs production [29, 30]. As a specific study limitation, we acknowledge that the study population will be affected by a geographic bias, and that this will limit the generalisation of the study results, as the same study conducted in countries with different levels of awareness and sensitivity about environmental protection and sustainability is likely to yield different results.

Finally, our hypothesis of only a 30% acceptance rate is highly conservative. The analysis of the actual acceptance rate according to sex, age, and other factors will elucidate this aspect. This enrolment rate could then represent a proxy metric of the “environmental awareness” of patients—a relevant social issue—allowing to further tailor the implementation of procedures aimed at reducing the environmental impact of CAs.

## Data Availability

Not applicable.

## Abbreviations

CA: Contrast agent
CT: Computed tomography
GBCA: Gadolinium-based contrast agent
GREENWATER: Reducing contrast agents’ residuals in hospital wastewater
ICA: Iodinated contrast agent
MRI: Magnetic resonance imaging
